# Dietary supplementation with postbiotics from *Bifidobacterium animalis* safely enhances multifunctional health in adult beagle dogs

**DOI:** 10.3389/fmicb.2026.1775856

**Published:** 2026-05-29

**Authors:** Mengyao Ma, Yuqiang Zhang, Huakai Wang, Weiwei Wang, Qianqian Chen, Ran Wang, Jianmin Wu, Jianmei Wang, Wanli Zhang, Qingyu Wen, Wei Xiong

**Affiliations:** 1 Food Laboratory of Zhongyuan, Luohe, China; 2 Key Laboratory of Precision Nutrition and Food Quality, Department of Nutrition and Health, China Agricultural University, Beijing, China

**Keywords:** gut microbiota, immune modulation, oxidative stress, postbiotics, short-chain fatty acids

## Abstract

**Introduction:**

Postbiotics are potential functional feed additives, but their effects on multiple health parameters in dogs remain largely unexplored.

**Methods:**

This study evaluated postbiotics from *Bifidobacterium animalis* in adult beagle dogs fed a basal diet supplemented with 0% (CONT), 0.05% (LDP), or 0.1% (HDP) postbiotics for 42 days.

**Results:**

No differences were observed in growth performance or routine blood parameters. However, postbiotic supplementation significantly increased serum IgA (LDP and HDP), IgM and IgG (HDP only), and reduced pro-inflammatory cytokines TNF-α and IL-6 (both groups), IL-1β (HDP only), while IL-10 was increased only in LDP. The HDP group showed lower malondialdehyde (MDA); SOD increased in both groups, while GSH-Px and CAT increased significantly only in HDP. Bone formation markers (PINP, OC) increased in HDP. Hair quality improved (smoother cuticle, higher glutamic acid, cysteine, proline) in both groups. Fecal acetic, propionic, and isovaleric acids increased in both groups; valeric acid increased only in HDP. Gut microbiota analysis revealed increased beneficial bacteria (such as *Limosilactobacillus*, *Lactobacillus*) and decreased potential pathogens (such as *Peptoclostridium*, *Streptococcus*).

**Discussion:**

Bifidobacterium animalis derived postbiotics safely enhance immune function, antioxidant status, bone metabolism, and hair quality in adult dogs, likely via gut microbiota modulation.

## 1 Introduction

As pets become more like humanized family members, owners seek precise and targeted nutritional strategies. These strategies aim to enhance animal health beyond basic maintenance. The gut microbiome is crucial for digestion, nutrient absorption, and energy metabolism. It also maintains intestinal barrier integrity, regulates immune responses, and defends against pathogens (Fang et al., 2025). Microbial dysbiosis is linked to canine inflammation, metabolic dysfunction, allergies, and immune impairment (Kayser et al., 2024). Therefore, regulating the gut microbiota is a key strategy for promoting health and preventing disease in dogs.

Probiotics, defined as live beneficial microorganisms, have been widely incorporated into canine diets to support gut health and immune function (Li et al., 2024). However, their practical application is limited by factors such as viability loss during processing and storage, variability among strains, and potential risks for immunocompromised individuals. In response, postbiotics—defined by the International Scientific Association for Probiotics and Prebiotics (ISAPP) as non-viable microbial cells, components, or metabolites that confer health benefits—have gained increasing attention (Pilla and Suchodolski, 2019; Sabahi et al., 2023; Salminen et al., 2022). Unlike probiotics, postbiotics offer advantages in stability, safety, and standardization, as their efficacy does not depend on cell viability (Salminen et al., 2022). Emerging evidence indicates that postbiotics exert multiple beneficial effects, including modulation of gut microbiota, stimulation of short-chain fatty acid (SCFA) production, enhancement of mucosal immunity, suppression of inflammatory pathways, and upregulation of tight-junction proteins (Sordillo et al., 2025). These properties position postbiotics as a promising avenue in functional pet nutrition and veterinary care.

Among beneficial microbes, *Bifidobacterium* species are well recognized for maintaining intestinal homeostasis and regulating immune and metabolic functions (Vinderola et al., 2022). Specifically, *Bifidobacterium animalis* has been studied as a canine probiotic, with reported benefits including improved fecal quality, enhanced intestinal barrier function, and potential dermatological and oral health effects. However, studies on postbiotic preparations derived from this species remain scarce (Yilmaz, 2024). Comprehensive *in vivo* data on their long-term safety, tolerability, and functional impacts on gut microbiota, immunity, and metabolism in healthy dogs are lacking (Zhao et al., 2024). Therefore, this study aimed to investigate the effects of dietary supplementation with *Bifidobacterium animalis*-derived postbiotics on body weight, blood biochemistry, immune and antioxidant status, bone metabolism markers, hair quality, SCFA profiles, and gut microbiota in healthy adult dogs. The findings are intended to provide experimental evidence supporting the application of postbiotics in functional dog foods and veterinary nutrition.

## 2 Materials and methods

### 2.1 Animals and experimental design

The animal experiment protocol of this study followed the Guide for the Care and Use of Laboratory Animals prepared by the Institutional Animal Care and Use Committee of China Agricultural University (Approval No. AW80215202-5-10). The postbiotics used in this study were derived from *Bifidobacterium animalis subsp. lactis* (8 × 10^11^ cells/g) and produced by Beijing Heyiyuan Biotechnology Co., Ltd. The product was produced via fermentation, followed by heat inactivation at 95 °C for 30 min, centrifugation, and freeze-drying. The resulting preparation consisted primarily of heat-inactivated bacterial cells and fermentation-derived metabolites. Although detailed compositional analysis (e.g., quantification of specific metabolites or cellular components) was not performed in this study, the product was manufactured using a standardized industrial process with quality control measures to ensure batch-to-batch consistency. This approach allowed for reproducible inclusion in the experimental diets.

After 7 days of adaptation, a total of 24 healthy adult Beagle dogs (equal numbers of male and female dogs; aged 16–20 months; weighing 7–8 kg) were randomly assigned to three groups (n = 8 per group): control (CONT), CONT supplemented with 0.05% postbiotics (LDP), and CONT supplemented with 0.1% postbiotics (HDP). Dogs were fed twice daily a total of 400 g of a basal diet that met all nutritional requirements according to the (National Research Council, 2006) recommendations for adult dogs. The nutritional composition of the diet is presented in Table 1. Before the experiment, all dogs were vaccinated against rabies and had received the standard pentavalent canine vaccine, and they underwent internal and external deworming treatments. None of the animals had been exposed to antibiotics or other microbiota-altering interventions for at least 4 weeks before the start of the study. Dogs were individually housed in sanitized cages with a relatively constant temperature (23–25 °C) and humidity (50–60%), and were provided with adequate lighting and water. Body weight and fecal consistency scores (using a standard 1–5 scale) were recorded on days 0, 21, and 42 of the experimental period.

**Table 1:** Dietary composition and nutritional level of basal diet.

Diet composition	%	Calculated nutrient level	%
Chicken powder	31.62	Moisture	9.00
Hulled barley	13.55	Crude protein	32.00
Field Pea	13.55	Crude fat	14.00
Rice	13.55	Crude fiber	8.00
Tapioca starch	6.32	Ash	9.00
Chicken fat	6.89		
Sweet potato granules	4.52		
Egg yolk powder	3.61		
Pet food compound seasoning	4.29		
Calcium carbonate	0.54		
Potassium chloride	0.36		
Choline chloride	0.36		
L-Lysine HCl	0.27		
Salt	0.18		
Vitamin E powder	0.05		
Mineral complexes and vitamins^1^	0.34		
Total	100.00		

^1^Mineral complexes and vitamins provided the following per kilogram of feed: vitamin A (14,500 IU), vitamin D_3_ (1,000 IU), vitamin E (156 IU), and vitamin B_1_ (32.0 mg), vitamin B_2_ (30.0 mg), vitamin B_3_ (120 mg), vitamin B_5_ (88.0 mg), vitamin B_6_ (13.0 mg), vitamin B_12_ (0.20 mg); Fe (FeSO4) 100 mg, Cu (CuSO4) 7.00 mg, Co (CoSO4) 1.00 mg, I (CaI2) 20.0 mg, Mn (MnSO4) 20.0 mg, Zn (ZnSO4) 68.0 mg, and Se (Na2SeO3) 0.50 mg.

### 2.2 Sample collection

On day 42, after an overnight fast, blood and hair samples were collected from all eight dogs per group, while fecal samples were collected from six dogs per group. All samples were immediately stored at −80 °C for later analysis. Blood samples were collected by shaving the hair on the dogs’ forelimbs to expose the saphenous vein (Wang et al., 2024). Serum was separated by centrifugation at 3,000 × g for 10 min at 4 °C and stored at −80 °C for biochemical and other analyses. Fresh feces were collected immediately after spontaneous defecation and stored at −80 °C for microbiota and SCFA analysis. Hair was trimmed from the back area using sterile scissors, placed in labeled envelopes, and stored for subsequent analysis.

### 2.3 Blood biochemistry analysis

Routine blood examinations were performed using an automated blood analyzer (Contac Co., Ltd., Qinhuangdao, Hebei, China). Serum superoxide dismutase (SOD), catalase (CAT), glutathione peroxidase (GSH-Px), malondialdehyde (MDA), tumor necrosis factor-*α* (TNF-α), interleukin-1β (IL-1β), interleukin-6 (IL-6), interleukin-10 (IL-10), osteocalcin (OC), type I procollagen N-terminal propeptide (PINP), and type II collagen C-terminal peptide (CTX-II) were measured by the enzyme-linked immunosorbent assay (ELISA) kit according to the manufacturer’s instructions (Shanghai Enzyme-Link Biotechnology Co., Ltd., Shanghai, China). Furthermore, serum immunoglobulin profiles (IgA, IgG, and IgM) were determined using turbidimetric immunoassay following the procedures provided by the manufacturers (Shanghai Enzyme-Link Biotechnology Co., Ltd., Shanghai, China).

### 2.4 Analysis of hair parameters

To assess hair microstructure, representative fibers were examined using scanning electron microscopy (SEM, SU8100, Hitachi, Tokyo, Japan), focusing on cuticle morphology. Meanwhile, approximately 50 mg of hair was collected into sterile tubes, placed immediately on dry ice, and later analyzed for free amino acid composition using ultra-high-performance liquid chromatography (UHPLC, APExBIO Technology LLC, Shanghai, China).

### 2.5 Determination of short-chain fatty acids

The quantitative analysis of SCFAs was conducted using gas chromatography (GC). Specifically, approximately 50 mg of sample was mixed with appropriate amounts of dilute sulfuric acid, diethyl ether, and an internal standard, and thoroughly homogenized. The mixture was then centrifuged at 12,000 × g for 10 min at 4 °C. The supernatant was collected and filtered through a 0.22 μm microporous membrane. The filtrate was then separated and detected using a GC system equipped with an Agilent DB-WAX UI column (30 m × 0.25 mm × 0.25 μm) and a flame ionization detector (FID). The column temperature program was set as follows: initial temperature 60 °C, maintained for 4 min; then increased to 180 °C at a rate of 6 °C/min; then increased to 200 °C at a rate of 20 °C/min and held for 10 min. The injection port and detector temperatures were both controlled at 250 °C, and nitrogen was used as the carrier gas at a flow rate of 1 mL/min. To complete the quantitative analysis, a series of concentration calibration solutions were prepared based on standard stock solutions of acetic acid, propionic acid, butyric acid, valeric acid, and isovaleric acid to establish a quantitative standard curve.

### 2.6 16S rRNA gene sequencing

Microbial genomic DNA was extracted using the E. Z. N. A.® Soil DNA Extraction Kit (Omega Biotek, Norcross, GA, USA). The extracted DNA was assessed for integrity and quality by 1% agarose gel electrophoresis, and its concentration and purity were determined using a NanoDrop™ 2000 spectrophotometer (Thermo Scientific, Waltham, MA, USA). DNA purity was deemed sufficient for subsequent experiments when the A260/280 ratio was greater than 1.8, and the A260/230 ratio exceeded 2.0. Subsequently, polymerase chain reaction (PCR) was performed using specific primers 341F (5’-CCTAYGGGRBGCASCAG-3′) and 806R (5’-GGACTACNNGGGGTATCTAAT-3′) to amplify the target fragment. The obtained PCR products were pooled and purified using the AxyPrep DNA Gel Extraction Kit (Axygen Biosciences, Union City, CA, USA) and accurately quantified using a Quantus™ fluorometer (Promega Corporation, Madison, WI, USA). The purified amplicons were used to construct sequencing libraries using the NEXTflex™ Rapid DNA Library Construction Kit (Bioo Scientific, Austin, TX, USA), and high-throughput sequencing was performed on an Illumina NovaSeq PE250 platform (Illumina, San Diego, CA, USA). The raw sequencing data were first demultiplexed and preliminarily quality-controlled using FastP (v0.20.0), and then paired-end sequences were merged using FLASH (v1.2.7) software. Furthermore, the UPARSE (version 7.1) algorithm was used to cluster operational taxonomic units (OTUs) at a 97% similarity level, while simultaneously identifying and removing chimeric sequences. To assess microbial diversity within samples, the ACE index, Chao1 index, Simpson index, and Shannon index were calculated using QIIME 2 (v2020.6). Principal coordinate analysis (PCoA) was performed based on the Bray–Curtis dissimilarity matrix to reveal differences in microbial community structure among different samples. Simultaneously, the relative abundance distribution of bacterial taxa was systematically assessed at multiple taxonomic levels at the genus level. Wilcoxon rank-sum test was used to compare the relative abundance of bacterial genera between groups, and *p*-values were adjusted for multiple comparisons using the Benjamini–Hochberg false discovery rate (FDR) method. An adjusted *p*-value (q-value) < 0.05 was considered statistically significant.

### 2.7 Statistical analysis

Statistical analysis of the experimental data was conducted using IBM SPSS Statistics software (Version 24.0; IBM Corporation, Armonk, New York, USA). For normally distributed parameters, a one-way analysis of variance (ANOVA) was employed to examine differences among the three experimental groups, followed by Tukey’s *post hoc* test for multiple comparison analysis. For non-normally distributed data, including gut microbiota *α*-diversity indices and relative abundances, the Kruskal-Wallis H test was used, followed by Dunn’s *post-hoc* test with Benjamini-Hochberg false discovery rate (FDR) correction for pairwise comparisons. The statistical significance level was set at *p* < 0.05.

## 3 Results

### 3.1 Growth performance

As shown in Table 2, body weight gradually increased over time in all groups. No significant differences in body weight were observed among the three groups, although numerically higher values were noted among the three groups. On day 21, the fecal score was significantly lower in the LDP and HDP groups than in the CONT group (*p* < 0.05).

**Table 2:** Effects of postbiotics on the growth performance of dogs.

Item	Day	CONT	LDP	HDP	*p-*value
Weight (kg)	0	7.42 ± 0.37	7.43 ± 0.41	7.57 ± 0.18	0.94
	21	7.51 ± 0.37	7.59 ± 0.38	7.85 ± 0.16	0.89
	42	7.64 ± 0.38	7.78 ± 0.38	8.12 ± 0.15	0.81
Fecal score	0	2.50 ± 0.00	2.50 ± 0.00	2.50 ± 0.00	1.00
	21	3.33 ± 0.08^b^	2.67 ± 0.08^a^	2.50 ± 0.00^a^	< 0.05
	42	2.83 ± 0.08	2.67 ± 0.08	2.67 ± 0.08	0.73

CONT, Control group; LDP, the CONT with 0.05% postbiotic supplementation; HDP, the CONT with 0.1% postbiotic supplementation. Data are presented as mean ± standard error. Different superscripts within the same row indicate significant differences (*p* < 0.05).

### 3.2 Routine blood parameters

As shown in Table 3, there were no differences in the routine blood parameters among the three groups; all measured values remained within the physiological reference ranges for healthy dogs, indicating that postbiotic administration did not cause any adverse hematological effects.

**Table 3:** Effects of postbiotics in the routine blood parameterts of dogs.

Item	CONT	LDP	HDP	*p-*value
Total white blood cells (10^9^/L)	13.96 ± 1.22	11.13 ± 0.60	10.87 ± 1.04	0.12
Lymphocyte ratio (%)	26.63 ± 2.14	28.42 ± 2.16	27.17 ± 3.32	0.96
Intermediate cell ratio (%)	7.70 ± 0.76	6.77 ± 0.42	6.81 ± 0.57	0.43
Granulocyte ratio (%)	55.05 ± 3.14	58.00 ± 2.55	58.76 ± 3.43	0.51
Lymphocytes (10^9^/L)	3.60 ± 0.29	3.15 ± 0.27	2.76 ± 0.26	0.08
Intermediate cells (10^9^/L)	1.07 ± 0.13	0.75 ± 0.07	0.73 ± 0.09	0.06
Granulocytes (10^9^/L)	7.89 ± 1.16	6.48 ± 0.53	6.58 ± 0.94	0.66
Total red blood cells (10^12^/L)	6.92 ± 0.21	6.83 ± 0.26	7.35 ± 0.20	0.34
Hemoglobin (g/L)	161.37 ± 4.28	159.50 ± 5.86	171.12 ± 3.75	0.40
Hematocrit (%)	44.96 ± 0.89	44.27 ± 1.40	47.30 ± 0.92	0.25
Mean corpuscular volume (fL)	65.15 ± 1.13	64.86 ± 0.61	64.43 ± 0.69	0.87
Hemoglobin content (pg)	23.33 ± 0.33	23.32 ± 0.14	23.28 ± 0.25	0.72
Hemoglobin concentration (g/L)	358.75 ± 4.10	359.75 ± 2.37	361.75 ± 3.47	0.98
Red cell distribution width SD (fL)	31.42 ± 0.77	31.41 ± 0.50	31.23 ± 0.57	0.96
Red cell distribution width CV (%)	15.15 ± 0.63	15.45 ± 0.48	16.05 ± 0.24	0.59
Total platelet count (10^9^/L)	312.12 ± 15.53	376.50 ± 18.28	313.37 ± 25.55	0.19
Mean platelet volume (fL)	10.83 ± 0.22	10.75 ± 0.26	10.175 ± 0.24	0.07
Platelet distribution width (%)	13.60 ± 0.49	13.87 ± 0.54	12.85 ± 0.58	0.12
Plateletcrit (%)	0.33 ± 0.02	0.40 ± 0.02	0.31 ± 0.02	0.06
Platelet–larger cell ratio (%)	34.37 ± 2.17	33.60 ± 2.43	28.41 ± 2.17	0.09

CONT, Control group; LDP, the CONT with 0.05% postbiotic supplementation, HDP, the CONT with 0.1% postbiotic supplementation. Data are presented as mean ± standard error.

### 3.3 Immunoglobulin levels

**Figure 1:**
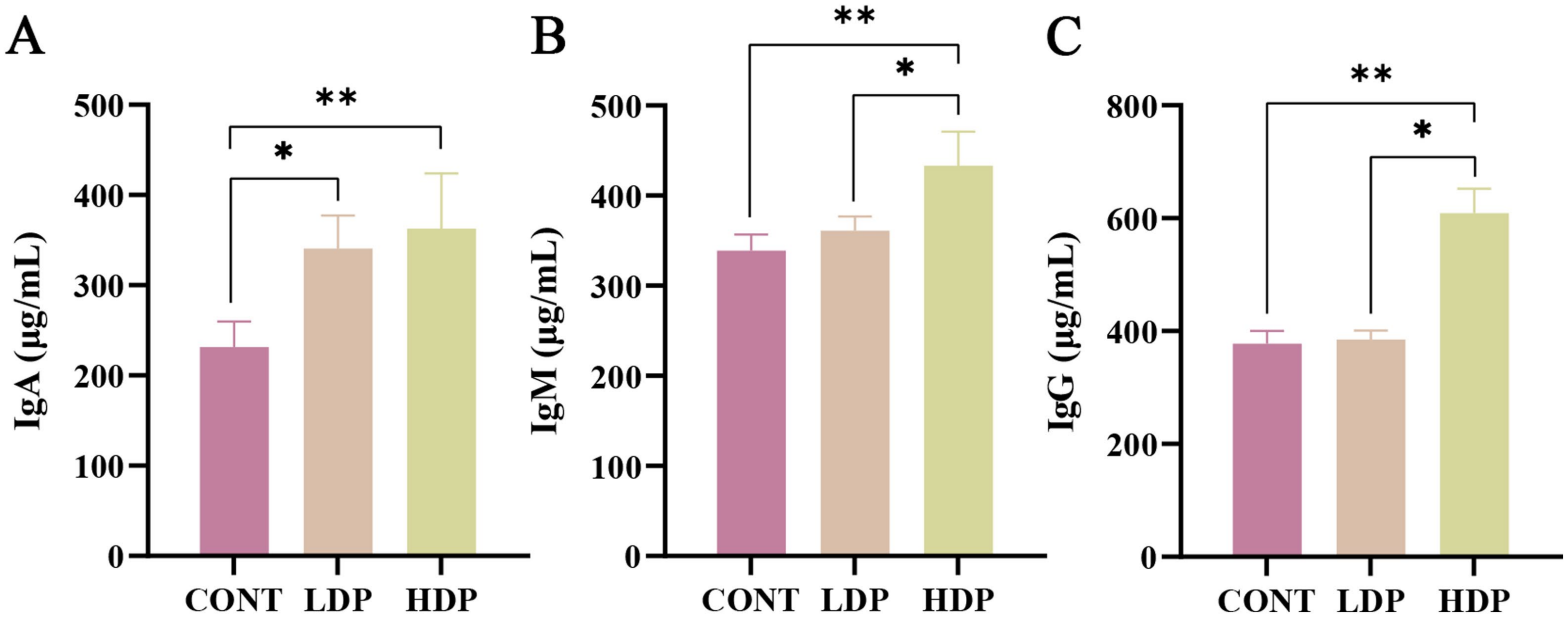
Effects of postbiotics on the immunoglobulin parameters in dogs. **(A)** IgA, **(B)** IgM, and **(C)** IgG. CONT, Control group, LDP, the CONT with 0.05% postbiotic supplementation, HDP: the CONT with 0.1% postbiotic supplementation. Data are presented as mean ± standard error. Statistical differences are indicated as *p < 0.05 and **p < 0.01.

The immunomodulatory effects of postbiotic supplementation are shown in Figure 1. Compared with the CONT group, both LDP and HDP groups exhibited significantly higher IgA concentrations (CONT: 231.65 μg/mL; LDP: 340.78 μg/mL; HDP: 362.65 μg/mL), representing increases of 47.11 and 56.56%, respectively (*p* < 0.05) (Figure 1A). For IgM, only the HDP group showed a significant increase (CONT: 338.76 μg/mL; HDP: 433.30 μg/mL), with an increase of 27.91% (*p* < 0.05), while the LDP group (360.87 μg/mL) did not differ significantly from CONT (Figure 1B). Similarly, IgG concentrations were significantly elevated only in the HDP group (CONT: 377.66 μg/mL; HDP: 608.72 μg/mL), corresponding to a 61.19% increase (*p* < 0.05), whereas the LDP group (384.81 μg/mL) showed no significant difference (Figure 1C). These results indicate that dietary supplementation with postbiotics enhances humoral immunity in dogs, with the high-dose (0.1%) supplementation exerting more pronounced effects.

### 3.4 Inflammatory cytokines

**Figure 2:**
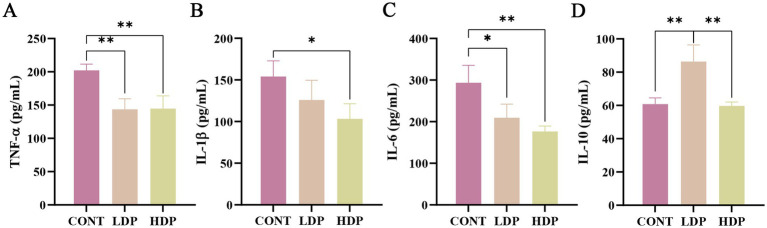
Effects of postbiotics on the levels of inflammatory cytokines in dogs **(A–D)**. CONT, Control group; LDP, the CONT with 0.05% postbiotic supplementation; HDP, the CONT with 0.1% postbiotic supplementation. Data are presented as mean ± standard error. Significant differences are indicated as *p < 0.05 and **p < 0.01.

The effects of postbiotic supplementation on inflammatory cytokines are shown in Figure 2. Compared with the CONT group, both LDP and HDP groups exhibited significantly lower TNF-*α* concentrations (CONT: 202.24 pg./mL; LDP: 143.64 pg./mL; HDP: 144.64 pg./mL), representing decreases of 28.98 and 28.48%, respectively (*p <* 0.05) (Figure 2A). For IL-1β, a significant reduction was observed only in the HDP group (CONT: 154.09 pg./mL; HDP: 103.31 pg./mL), with a decrease of 32.95% (*p* < 0.05), while the LDP group (125.90 pg./mL, 18.29% decrease) did not differ significantly from CONT (Figure 2B). IL-6 concentrations were significantly reduced in both postbiotic-supplemented groups (CONT: 293.54 pg./mL; LDP: 209.36 pg./mL; HDP: 176.45 pg./mL) compared with the CONT group (*p <* 0.05) (Figure 2C). In contrast, IL-10 levels were significantly increased only in the LDP group (CONT: 60.76 pg./mL; LDP: 86.41 pg./mL), with an increase of 42.21% (*p <* 0.05), while the HDP group (61.90 pg./mL) showed no significant difference from CONT (Figure 2D). These results indicate that dietary postbiotic supplementation can modulate inflammatory balance in dogs, contributing to improved immune homeostasis and reduced inflammatory burden.

### 3.5 Oxidative stress biomarkers

**Figure 3:**
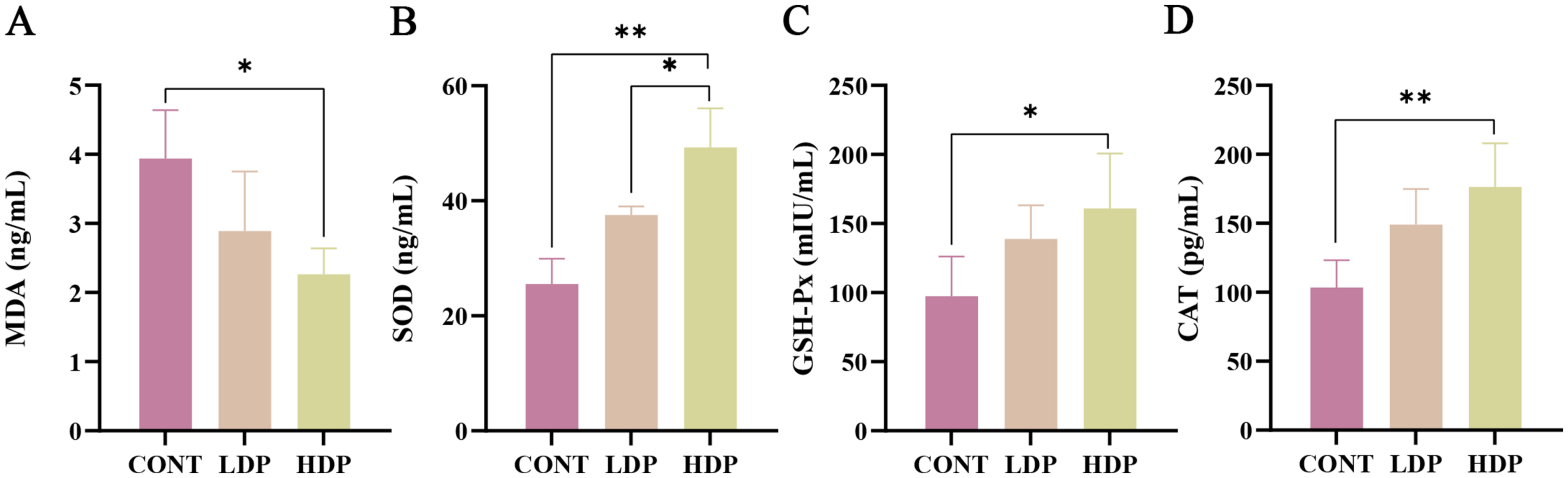
Effects of postbiotics on the levels of oxidative stress-related indicators in dogs. **(A)** MDA, **(B)** SOD, **(C)** GSH-Px, and **(D)** CAT. CONT, Control group; LDP, the CONT with 0.05% postbiotic supplementation; HDP, the CONT with 0.1% postbiotic supplementation. Data are presented as mean ± standard error. Significant differences are indicated as *p < 0.05 and **p < 0.01.

The effects of postbiotic supplementation on serum antioxidant markers are shown in Figure 3. Compared with the CONT group, MDA concentrations were significantly reduced in both LDP and HDP groups (CONT: 3.94 ng/mL; LDP: 2.89 ng/mL; HDP: 2.27 ng/mL), representing decreases of 26.57 and 42.47%, respectively (*p <* 0.05) (Figure 3A). Conversely, SOD concentrations (Figure 3B) increased from 25.55 ng/mL in the CONT group to 36.86 ng/mL in the LDP group and 49.25 ng/mL in the HDP group, corresponding to increases of 44.29 and 92.81%, respectively (*p <* 0.05). Similarly, GSH-Px level (CONT: 97.44 mIU/mL; LDP: 138.89 mIU/mL; HDP: 161.01 mIU/mL) and CAT concentration (CONT: 103.52 pg./mL; LDP: 149.02 pg./mL; HDP: 176.38 pg./mL) were elevated in both postbiotic-supplemented groups, with the HDP group reaching statistical significance (*p <* 0.05), while the LDP group showed a numerical but non-significant increase. These results indicate that dietary postbiotic supplementation effectively enhances serum antioxidant capacity in dogs.

### 3.6 Bone metabolism biomarkers

**Figure 4:**
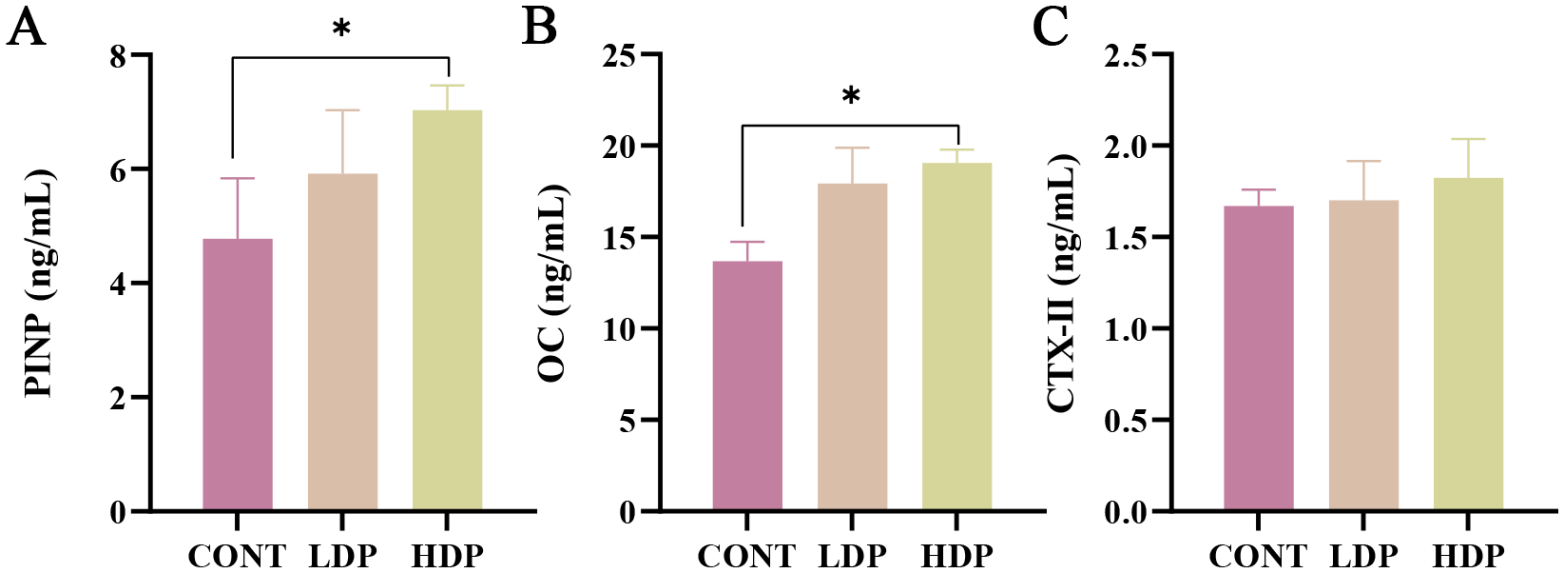
Effects of postbiotics on the levels of markers related to canine bone metabolism. **(A)** PINP, **(B)** OC, and **(C)** CTX-II. CONT, Control group; LDP, the CONT with 0.05% postbiotic supplementation; HDP, the CONT with 0.1% postbiotic supplementation. Data are presented as mean ± standard error. Significant differences are indicated as *p < 0.05.

The effects of postbiotic supplementation on bone metabolism markers are shown in Figure 4. Compared with the CONT group, PINP (CONT: 4.78 ng/mL; LDP: 5.92 ng/mL; HDP: 7.03 ng/mL) and OC (CONT: 13.68 ng/mL; LDP: 17.93 ng/mL; HDP: 19.06 ng/mL) concentrations were increased, with the HDP group reaching statistical significance (*p <* 0.05). In contrast, no significant differences in CTX-II concentrations were observed among the groups (CONT: 1.68 ng/mL; LDP: 1.81 ng/mL; HDP: 1.92 ng/mL) (Figure 4C). These results indicate that dietary postbiotic supplementation may promote bone formation in dogs, as evidenced by increased PINP and OC levels, without affecting bone resorption marker CTX-II.

### 3.7 Hair microstructural observation

**Figure 5:**
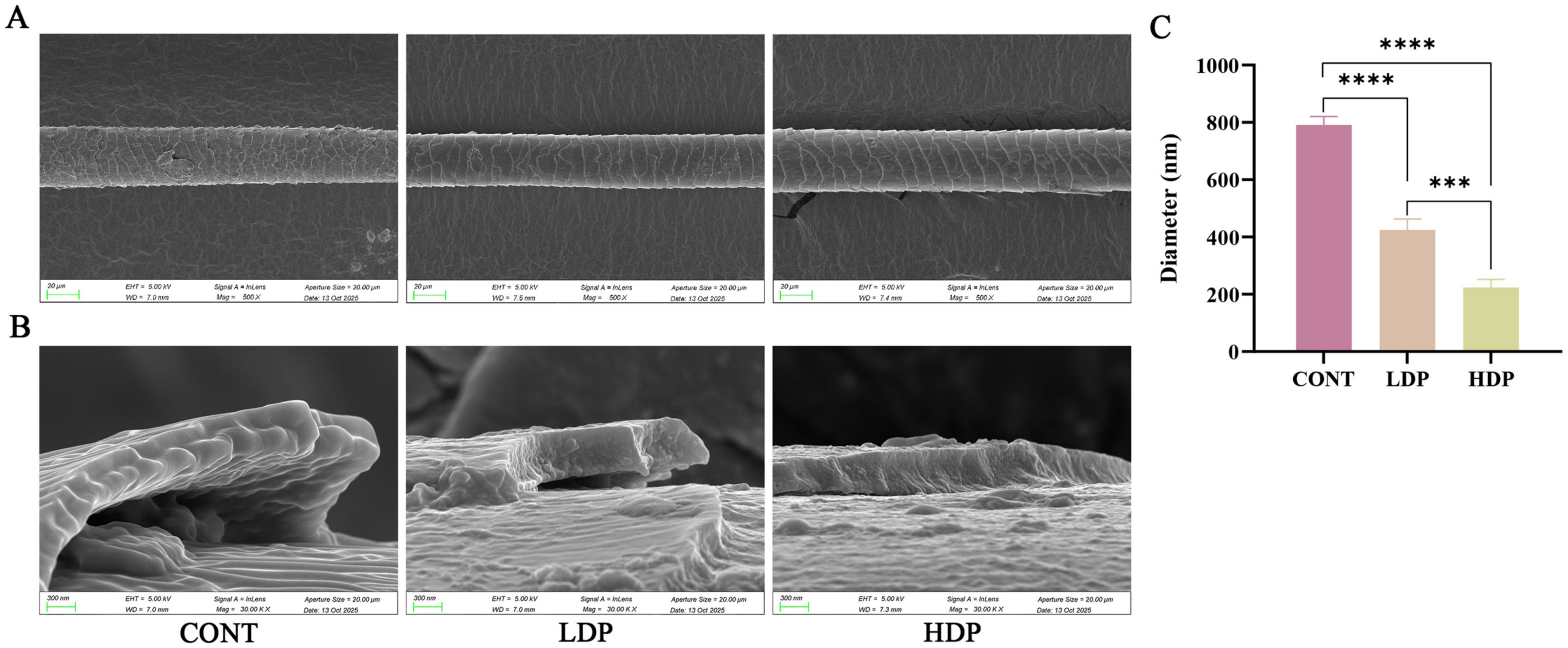
Effects of postbiotics on canine hair structural characteristics. **(A)** SEM images show the regularity of hair in the CONT, LDP, and HDP groups; **(B)** SEM images showing hair scale thickness in the CONT, LDP, and HDP groups; **(C)** Quantitative comparisons of hair diameter among the CONT, LDP, and HDP groups, respectively. CONT, Control group; LDP, the CONT with 0.05% postbiotic supplementation; HDP, the CONT with 0.1% postbiotic supplementation. Data are presented as mean ± standard error. Significant differences are indicated as ***p < 0.001 and ****p < 0.0001.

As shown in Figure 5, hair shafts displayed pronounced surface deterioration, characterized by coarse textures, evident cracking, lifted and fragmented cuticle edges, and disordered scale arrangement in the CONT group. In contrast, hair surfaces were smoother, with fewer surface discontinuities and more compact, regularly aligned cuticle layers in the LDP and HDP groups. Quantitative results showed that the LDP and HDP groups had lower hair diameter compared with the CONT group (*p <* 0.05, Figure 5C). These ultrastructural improvements suggest that postbiotic supplementation contributes to enhanced cuticle integrity and overall hair-shaft quality.

### 3.8 Hair amino acid profile

As shown in Table 4, compared with the CONT group, the LDP group and the HDP group increased the Glutamic acid, Cystine, and Proline contents (*p <* 0.05). The results indicate that postbiotics can significantly increase amino acids closely related to keratin structure and hair fiber integrity, exhibiting a certain dose-related regulatory effect.

**Table 4:** Effects of postbiotics on the amino acid levels (mg/g) in canine hair.

Item	CONT	LDP	HDP	*p-*value
Aspartic acid	55.13 ± 0.65	55.67 ± 0.71	54.86 ± 0.83	0.76
Threonine	54.04 ± 1.47	56.57 ± 0.90	54.43 ± 0.87	0.36
Serine	80.10 ± 1.24	83.8 ± 1.27	85.26 ± 0.37	0.10
Glutamic acid	145.92 ± 2.01^b^	155.40 ± 2.33^a^	156.68 ± 1.44^a^	< 0.05
Glycine	39.04 ± 1.08	41.52 ± 0.43	38.94 ± 1.61	0.53
Alanine	26.65 ± 1.34	26.97 ± 0.63	26.73 ± 1.02	0.95
Cystine	79.78 + 0.74^b^	88.79 + 0.67^a^	91.21 + 0.66^a^	< 0.05
Valine	32.99 ± 1.18	34.87 ± 0.76	34.26 ± 1.24	0.67
Methionine	7.93 ± 0.19	8.45 ± 0.49	7.92 ± 0.08	0.73
Isoleucine	20.06 ± 0.31	21.21 ± 0.29	20.18 ± 0.22	0.13
Leucine	54.17 ± 0.08	57.01 ± 1.43	55.97 ± 0.60	0.55
Tyrosine	37.68 ± 1.92	39.09 ± 0.56	36.88 ± 1.52	0.74
Phenylalanine	25.03 ± 0.92	25.65 ± 0.80	24.65 ± 0.79	0.64
Histidine	13.80 ± 0.51	14.08 ± 0.32	14.22 ± 0.50	0.79
Lysine	37.75 ± 0.46	40.08 ± 1.24	39.22 ± 1.00	0.64
Arginine	77.20 ± 2.23	79.87 ± 0.75	77.99 ± 0.65	0.51
Proline	60.80 ± 2.51^c^	74.60 ± 2.38^b^	83.14 ± 1.22^a^	<0.05

CONT, Control group; LDP, the CONT with 0.05% postbiotic supplementation; HDP, the CONT with 0.1% postbiotic supplementation. Data are presented as mean ± standard error. Different superscripts within the same row indicate significant differences (*p* < 0.05).

### 3.9 Fecal short-chain fatty acids

**Figure 6:**
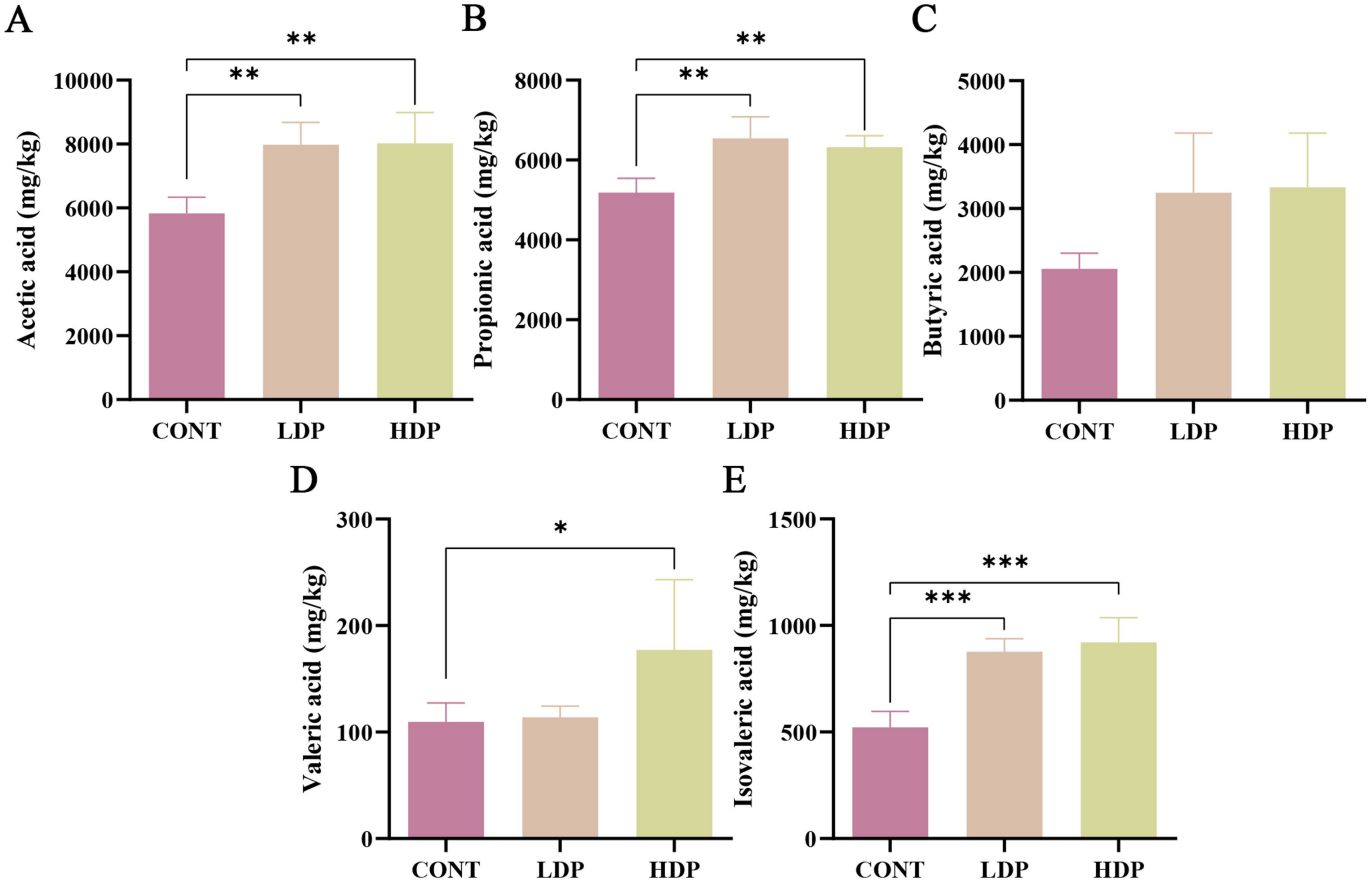
Effects of postbiotics on the SCFA content in canine feces. **(A)** Acetic acid, **(B)** propionic acid, **(C)** butyric acid, **(D)** valeric acid, and **(E)** isovaleric acid. CONT, Control group; LDP, the CONT with 0.05% postbiotic supplementation; HDP, the CONT with 0.1% postbiotic supplementation. Data are presented as mean ± standard error. Significant differences are indicated as *p < 0.05, **p < 0.01 and ***p < 0.001.

The concentrations of SCFAs in the different groups are presented in Figure 6. Compared to the control group, both LDP and HDP groups exhibited significantly higher (*p <* 0.05) concentrations of acetic acid (CONT: 5830.05 mg/kg; LDP: 7977.99 mg/kg; HDP: 8019.46 mg/kg), propionic acid (CONT: 5182.62 mg/kg; LDP: 6546.60 mg/kg; HDP: 6325.76 mg/kg), and isovaleric acid (CONT: 521.30 mg/kg; LDP: 876.78 mg/kg; HDP: 920.88 mg/kg). For valeric acid, only the HDP group (177.10 mg/kg) showed a significant increase compared to the CONT group (109.64 mg/kg) (*p <* 0.05), with an increase of 61.52%, while the LDP group (113.80 mg/kg) did not differ significantly. In contrast, no significant differences were observed among the groups in butyric acid concentrations (CONT: 2056.44 mg/kg; LDP: 3294.19 mg/kg; HDP: 3331.91 mg/kg), although a notable numerical increase was detected in both treatment groups. Furthermore, isobutyrate and hexanoate were not detected in any of the experimental groups.

### 3.10 Fecal microbiota community of dogs

**Figure 7:**
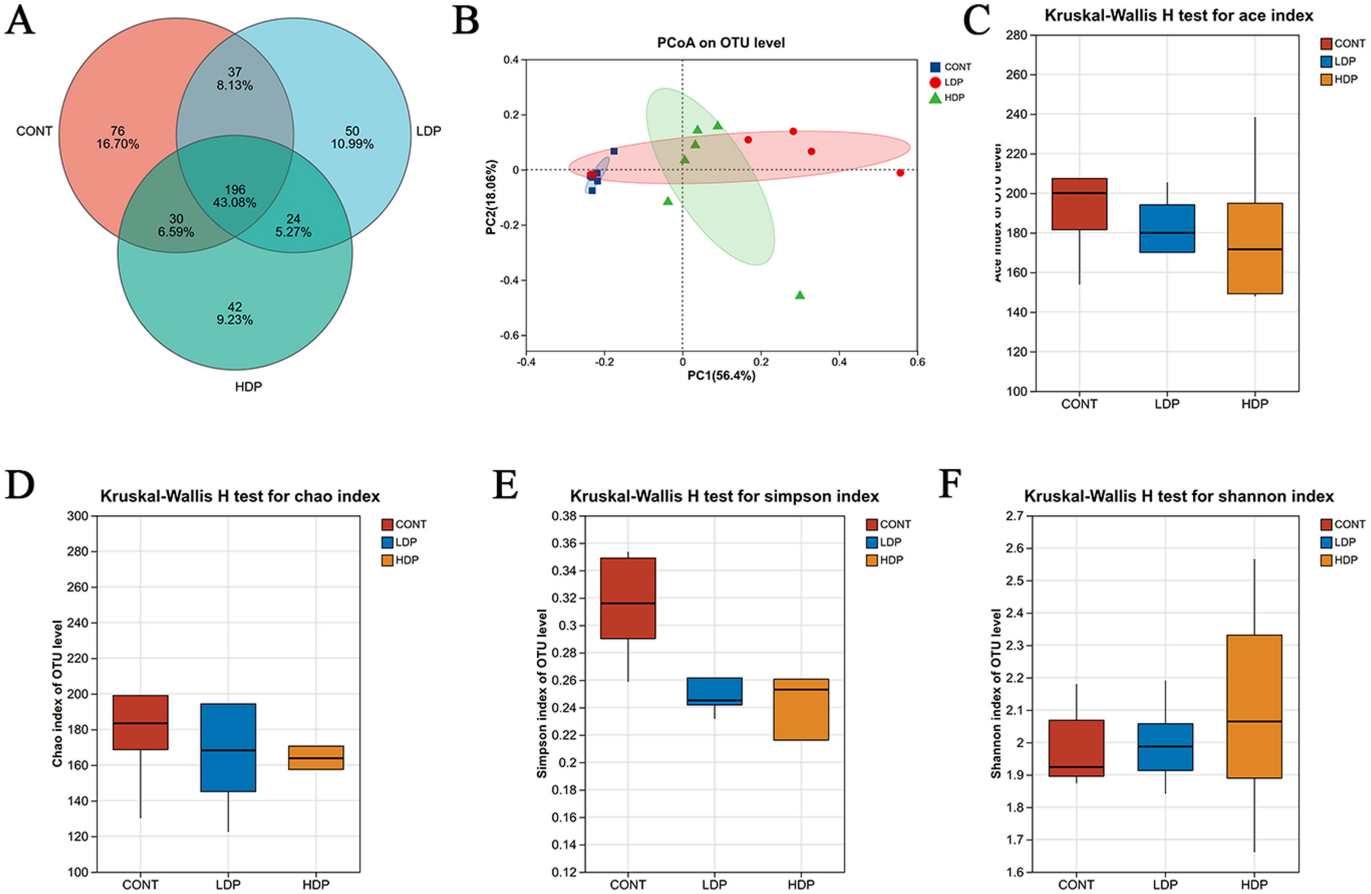
Effects of postbiotics on the composition of fecal microbiota and diversity in dogs. **(A)** Venn diagram of OTUs; **(B)** Principal coordinate analysis (PCoA) of gut microbiota; **(C–F)** Box plots showing *α*-diversity indices (Ace, Chao, Simpson, Shannon). The Kruskal–Wallis H test was used to compare the three groups, followed by Dunn’s *post-hoc* test for pairwise comparisons. No significant differences were observed (*p* > 0.05 for all). CONT, Control group; LDP, the CONT with 0.05% postbiotic supplementation; HDP, the CONT with 0.1% postbiotic supplementation. Data are presented as mean ± standard error.

**Figure 8:**
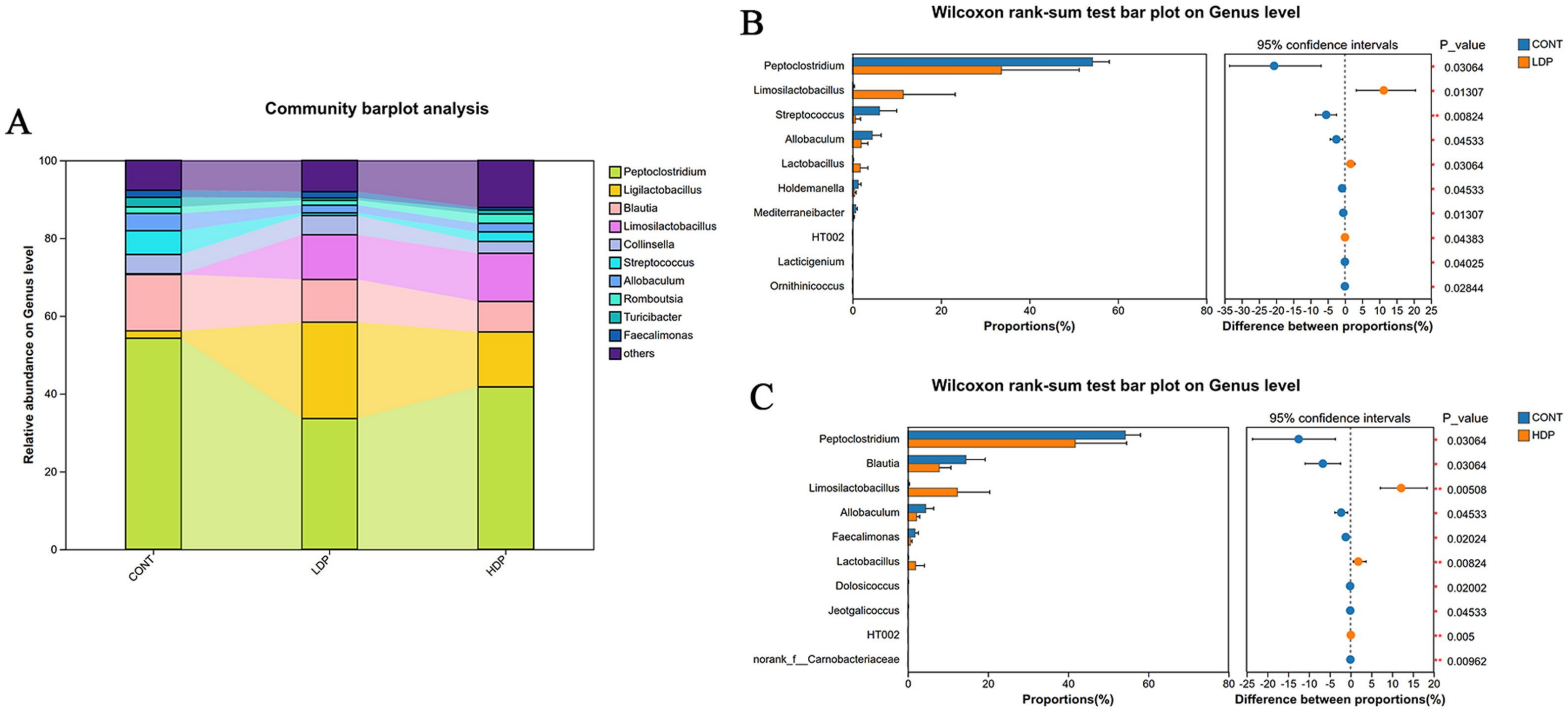
Effects of postbiotic supplementation on canine gut microbiota composition. **(A)** Relative abundance of gut microbiota at the genus level; **(B)** Wilcoxon rank-sum test between the CONT and LDP groups; **(C)** Wilcoxon rank-sum test between the CONT and HDP groups. Significance levels were determined using the Wilcoxon rank-sum test followed by Benjamini–Hochberg FDR correction for multiple comparisons. Only genera with an adjusted *p*-value (*q*-value) < 0.05 are shown as significant. CONT, Control group; LDP, the CONT with 0.05% postbiotic supplementation, HDP, the CONT with 0.1% postbiotic supplementation. Data are presented as mean ± standard error.

The Venn diagram showed that a total of 196 shared OTUs were present in the three groups, while CONT, LDP, and HDP groups contained 76, 50, and 42 different OTUs, respectively (Figure 7A). The results of PCoA displayed a distinct separation among different groups, indicating that postbiotic supplementation altered the gut microbiota composition in dogs (Figure 7B). However, there was no difference in the *α*-diversity indexes, including ACE, Shannon, Chao, and Simpson indexes, among the three groups (Figures 7C–F). At the genus level, *Peptoclostridium* and *Ligilactobacillus* were the dominant bacterial communities, followed by *Blautia*, *Limosilactobacillus*, *Collinsella*, and *Streptococcus* (Figure 8A). Compared with the CONT group, the LDP group decreased the relative abundance of *Peptoclostridium*, *Streptococcus*, *Allobaculum*, *Holdemanella*, *Mediterraneibacter*, *Lacticigenium*, and *Ornithiicoccus*, while increasing the relative abundance of *Limosilactobacillus*, *Lactobacillus*, and *HT002* (Figure 8B); the HDP group decreased the relative abundance of *Peptoclostridium*, *Blautia*, *Allobaculum*, *Faecalimonas*, *Dolosicoccus*, *Jeotgalicoccus*, and *norank_f_Carmobacteriaceae*, while increasing the relative abundance of *Limosilactobacillus*, *Lactobacillus*, and *HT002* (Figure 8C).

## 4 Discussion

**Figure 9:**
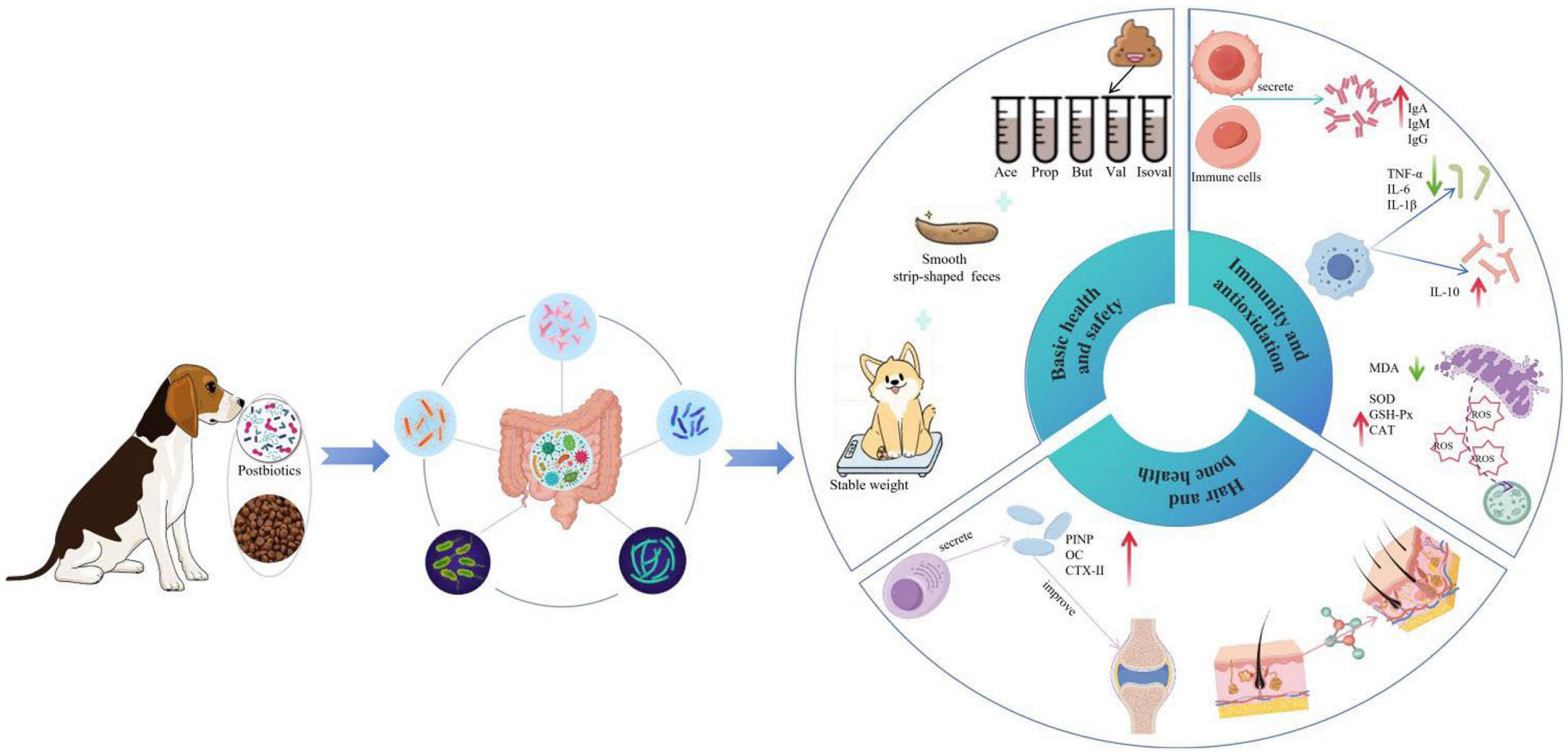
Proposed mechanism of postbiotics in improving health status in dogs through modulation of gut microbiota.

While research on the efficacy of *Bifidobacterium animalis* is extensive (Araújo et al., 2022), studies investigating its postbiotic preparations in pets remain scarce. The present study investigated the effects of dietary supplementation with *Bifidobacterium animalis*-derived postbiotics on growth performance, systemic physiology, hair quality, and gut microbiota in adult dogs. The results demonstrate that postbiotic supplementation exerts multiple health benefits through a gut-systemic axis, modulating the gut microbiota and its metabolites, which in turn influence systemic immune function, antioxidant status, bone metabolism, and hair quality (Figure 9). Importantly, no adverse effects on growth performance, fecal consistency, or hematological parameters were observed, confirming the safety and tolerability of the postbiotic preparation, consistent with previous reports on inactivated microbial products (Yan et al., 2024).

The gut microbiota serves as the primary interface through which postbiotics initiate their systemic effects. In the present study, postbiotic supplementation modulated the composition of the fecal microbiota, increasing the abundance of potentially beneficial genera such as *Limosilactobacillus*, *Lactobacillus*, and *HT002*, while reducing the relative abundance of several taxa associated with opportunistic pathogenicity or metabolic dysfunction, including *Peptoclostridium*, *Streptococcus*, and *Blautia* (Luo et al., 2016; Weiser et al., 2018; Hu et al., 2023; Sheikh et al., 2024). These shifts in microbial community structure were accompanied by significant increases in fecal concentrations of acetic acid, propionic acid, and valeric acid—key SCFAs that serve as both energy substrates for colonocytes and signaling molecules with systemic reach (Parada Venegas et al., 2019). Although butyric acid also showed a marked numerical increase, the difference did not reach statistical significance, which may be attributable to high inter-individual variability and the limited sample size, indicating a potential risk of type II error; therefore, this result should be interpreted with caution. The enhanced SCFA production suggests that postbiotics not only alter microbial composition but also promote its metabolic activity, thereby amplifying their physiological impact.

SCFAs and other microbial metabolites are increasingly recognized as mediators of gut-systemic crosstalk, influencing immune function, oxidative stress, and tissue metabolism (Sordillo et al., 2025). In line with this, postbiotic supplementation significantly enhanced systemic immune parameters, as evidenced by increased serum IgA, IgM, and IgG concentrations. This immunostimulatory effect likely arises from microbial components (e.g., cell wall fragments, lipoteichoic acids) and metabolites that interact with pattern recognition receptors on immune cells, a mechanism well documented for postbiotics derived from *Lactobacillus* and *Bifidobacterium* strains (Hosseini et al., 2024; Aguilar-Toalá et al., 2018). Concurrently, postbiotics modulated the inflammatory cytokine balance, reducing pro-inflammatory mediators (TNF-*α*, IL-1β, IL-6) while elevating the anti-inflammatory cytokine IL-10 in a dose-dependent manner. These findings align with previous reports that postbiotics suppress NF-κB signaling and subsequent inflammatory cascades (Zhong et al., 2022; Duysburgh et al., 2024). The non-linear response of IL-10—elevated only in the LDP group—may reflect a reduced feedback demand for this cytokine under the more pronounced pro-inflammatory suppression achieved with the higher dose.

Oxidative stress is intrinsically linked to inflammation and immune dysfunction, and contributes to the deterioration of skin and coat condition in dogs (Liu et al., 2021). Postbiotic supplementation strengthened the systemic antioxidant defense, as shown by increased activities of SOD, GSH-Px, and CAT, and reduced MDA levels—a marker of lipid peroxidation. These enzymes constitute the primary antioxidant system protecting cells from oxidative damage (Bogacka et al., 2024). The observed enhancement is consistent with studies demonstrating that postbiotic metabolites can upregulate endogenous antioxidant enzymes and scavenge reactive oxygen species (Ciltas et al., 2023; Asadi et al., 2024). The coordinated improvement in both immune and antioxidant parameters suggests that postbiotics exert a broad, integrated effect on systemic homeostasis.

Beyond immune and antioxidant effects, postbiotic supplementation influenced two clinically relevant outcomes in dogs: bone metabolism and hair quality. Although research on postbiotics and joint health in companion animals is limited, emerging evidence suggests that microbial metabolites, particularly propionate and butyrate, may contribute to cartilage homeostasis and bone mineral metabolism (Holmberg et al., 2024; Hao et al., 2024). In the present study, postbiotic supplementation increased serum PINP and OC—markers of bone formation—without altering CTX-II, a bone resorption marker. This selective enhancement of bone formation markers suggests a potential anabolic effect on skeletal tissue, warranting further investigation in long-term or disease-model studies, particularly given the relevance of skeletal degeneration in aging dogs.

Hair quality, an important indicator of overall health in companion animals, was also improved by postbiotic supplementation. Dogs receiving postbiotics exhibited smoother hair cuticles, reduced cuticle thickness, and elevated levels of keratin-associated amino acids (glutamic acid, cysteine, proline). These changes likely reflect improved nutrient metabolism and delivery to hair follicles, potentially mediated by enhanced gut function and systemic metabolic status (De Almeida et al., 2023). The observed improvements align with the growing recognition of a “gut-skin axis,” whereby gut microbiota and their metabolites influence skin and coat health (Feng, 2024).

Taken together, these findings support a model in which dietary postbiotics derived from *Bifidobacterium animalis* act primarily on the gut microbiota, enhancing its metabolic output (notably SCFAs). These microbial metabolites and components then enter the systemic circulation, where they modulate immune function, reduce oxidative stress, and exert trophic effects on distal tissues including bone and hair follicles. This integrated, multi-system response underscores the potential of postbiotics as functional nutritional supplements that promote canine health through the gut-systemic axis.

Several limitations should be acknowledged. First, the postbiotic preparation used in this study was not chemically characterized beyond its origin and manufacturing process. Therefore, we cannot attribute the observed effects to specific metabolites or cellular components. Second, the sample size for fecal analysis was relatively small (*n* = 6 per group), which may have limited statistical power for detecting differences in butyric acid and some microbial taxa. Future studies should combine metabolomic profiling with larger cohorts to identify the active principles underlying the beneficial effects of *B. animalis*-derived postbiotics.

## 5 Conclusion

This 42-day randomized controlled trial demonstrated that dietary supplementation with *Bifidobacterium animalis*-derived postbiotics is safe and well tolerated in healthy adult Beagle dogs. Postbiotic supplementation modulated the composition and metabolic activity of the gut microbiota, increasing the production of short-chain fatty acids. These changes were associated with a cascade of systemic effects, including enhanced humoral and cellular immune responses, strengthened antioxidant capacity, improved bone formation markers, and better hair quality. Collectively, these findings provide evidence that postbiotics exert multiple health benefits through the gut-systemic axis, supporting their application as functional dietary components in canine nutrition. Future studies should investigate the long-term effects of postbiotic supplementation and explore the specific metabolites responsible for the observed physiological improvements.

## Data Availability

The original contributions presented in the study are publicly available. This data can be found at: https://www.ncbi.nlm.nih.gov/bioproject/PRJNA1462474.
